# Illustrations of the Elementary Forms of Disease

**Published:** 1834-07-01

**Authors:** 


### ILLUSTRATIONS OF THE ELEMENTARY FORMS OF DISEASE. By *Robert Carswell*, M.D., &c. &c.

[Fasciculus fifth.—SOFTENING.]

THE term softening is employed to designate a diminution of the natural and healthy consistence of organs. It is only of late years that the lesion has been investigated as a special morbid condition, although it deserves, upon every account, the attentive consideration of the scientific physician.

Dr. Carswell first enumerates the pathological phenomena which precede the lesion, and on these phenomena he founds his divisions of the latter. Perhaps this is not the most natural order; it undoubtedly possesses some advantages, and Dr. Carswell's arrangement is so firmly grafted on it, and so intimately intertwined with it, that we cannot adopt any other method of laying his descriptions and his views before our readers.

Omitting some general introductory matter, we may state that our author divides softening into two generic kinds; that which occurs during life—and that which takes place after death. The first he subdivides into softening from inflammation—softening from obliteration of arteries—softening from modifications of nutrition. The second, or the cadaveric softening, is arranged under the heads of softening from the chemical action of the gastric juice—and, softening from maceration and putrefaction. But he limits his observations in the present fasciculus to the softening which results from inflammation—from obliteration of arteries—and from the chemical action of the gastric juice. These are the most important varieties of the alteration, and we repeat that they are worthy of the attention of our readers.

### Softening from Inflammation.

We confess that we are startled with the opening of this section. The boldness of the enunciation of the fact is only excelled by the confidence of the application of the theory. Let our readers judge.

"It may be laid down," says Dr. Carswell, "as a general rule, that every organ or tissue affected with acute inflammation, undergoes at the same time a diminution of consistence. The principle on which this change is effected is obvious, viz. cessation of the circulation, and, consequently, of nutrition, in the inflamed tissue. This state of the circulation is generally admitted to constitute one of the essential local characters of inflammation. It occurs subsequently to the accumulation of the blood in the capillaries, is always preceded by the effusion of serosity, and, in general, is accompanied by the formation and deposition of coagulable lymph and pus. Under these circumstances a supply of assimilable materials is cut off, and the function of nutrition is entirely suspended. A diminution in the consistence of the tissue thus situated follows, the degree of which will vary with the duration of the suspended function, and the degree of cohesion possessed by the tissue in its normal condition."

We are really not prepared to acquiesce in the declaration, that every tissue acutely inflamed is less consistent than when healthy. The whole passage is so involved that it is difficult to determine the precise meaning of our author. It would appear that the softening occurs at that period of inflammation which follows the effusion of serosity, and is generally accompanied by the formation of lymph and pus. It will not escape the observation of our readers, that Dr. Carswell takes for granted the truth of that *theory* of inflammation, of which cessation of the capillary circulation is the essence—a theory to which, to say the least, there are objections. And yet it does not appear necessary to his view, because the softening results from the effusion of fluids, serum or pus, which are actual circumstances, and independent of theory altogether. But it is not, after all, on such effusion that the softening in question depends, but on "cessation of the circulation and consequently of nutrition in the inflamed tissue." Now it is not by any means obvious to us why softening should be a necessary consequence of diminution or cessation of nutrition. If bone be inflamed and phosphate of lime be deposited in its interstitial texture in consequence of inflammation, nutrition may be interfered with, and yet the reverse of softening takes place. Again, if the cellular tissue be infiltrated with serum or with pus, it may surely be softened independently of any peculiar cessation of nutrition. We have formerly remarked the singular disposition on the part of pathological writers to indulge in speculative reasoning—in speculative reasoning on that which is ostentatiously, and indeed very properly advanced as a science of simple facts. We think that this tendency may be traced to the French school—one distinguished by the liveliness of its fancy, and the hastiness and boldness of its generalization, rather than by its strict adherence to inductive reasoning.

Our habits and our taste incline us to pass at once to more solid matter, and we therefore proceed to the physical characters of inflammatory softening. Dr. Carswell commences with those which are noticed in the brain.

*Inflammatory Softening in the Brain.* The degree may vary from a slight diminution of the natural consistence of the organ to that of cream or milk. The first stage, or, perhaps, more strictly speaking, the first degree, is often so inconsiderable as to be hardly perceptible to the touch or sight. To detect it a very careful examination is required. In the second stage the diminution of consistence may be recognized at once by the change of form to which it gives rise. The cerebral substance sinks by its own weight beneath the level of the cut surface; and prominent parts, such as the thalami, corpora striata, and convolutions, become more or less flattened. In the third stage a solution of continuity has been effected by the separation and partial removal of the softened cerebral substance. It is now of the consistence of cream or milk, contained in an excavation of variable extent, situated in the substance of the brain, or confined between the membranes and convolutions of this organ. All these degrees of softening may be met with in the same, or different portions of the brain at the same time.

Softening varies greatly in extent. It may occur in one portion only of the brain, or in several at once—in the cerebrum and cerebellum; but it is rarely observed in the latter, unless it be also present in the former.

The colour of inflammatory softening of the cerebral substance also presents considerable variety. This principally depends on the quantity of blood contained in the affected part, on changes which this fluid undergoes some time after its accumulation or effusion, and on the presence of serosity or pus.

"Redness and vascularity are, in general, greater in the first than in the second stage of softening, but the degree and the extent of either greatly depend on the quantity of blood in the cerebral vascular system. In some cases the redness and vascularity extend to a considerable distance beyond the softened part, diminishing gradually in intensity; in others they are limited to the immediate vicinity of the softening. The vascularity of the softened cerebral substance may be ramiform or punctiform, but it has more frequently a hemorrhagic character. When this substance is divided, it presents a number of red points, streaks, or patches, produced by the blood accumulated in the veins, or by the effusion of this fluid. In some cases the effused blood is small in quantity compared with the extent of the softening; in others it pervades the whole of the softened substance, and sometimes presents the appearance of hemorrhagic apoplexy. The redness, vascularity, and hemorrhagic character of inflammatory softening, are seldom so conspicuous as when this lesion occupies the brown substance, more especially of the corpora striata and thalami."

Inflammatory softening of the cerebral substance is not always accompanied with these changes of colour; for sometimes the latter may be natural, or even paler than it should be. Pale softening in the white and brown substance of the brain is a frequent occurrence in hydrocephalus—an occasional one in those fevers in which the brain is primarily or secondarily affected. In such cases the vascular system of the brain contains but little blood, and its membranes are infiltrated with serosity. When the pale softening is the consequence of meningitis, it is often confined to the brown substance of the convolutions, and is frequently detected while removing the pia mater, in consequence of portions of this substance being carried away with the bloodvessels which connect it with this membrane.

When redness and softening arise from the presence of effused blood, the softening is of recent date. But when the colour is brown, yellow, and orange, either separate or combined, and occupying either the softened substance, the part of the brain contiguous to it, or both at the same time, the disease has existed for weeks, or even months. These varieties of colour are not observed unless the softening has been accompanied by sanguineous effusion, and originate in changes taking place in the effused blood, changes analogous to those observed in sanguineous extravasation in the subcutaneous cellular tissue after injury.

A pale yellow straw-coloured tinge of the softened cerebral substance arises from the presence of pus, but is rarely noticed, unless the softened substance be in contact with the membranes of the brain. Serosity produces not only pale softening, but likewise communicates a peculiar glossy, albuminous aspect to the alteration. When softening of the cerebral substance is accompanied by an increase of bulk, which is seldom great, it is chiefly owing to the presence of serosity.

Similar characters to those described above are displayed by inflammatory softening of the cerebellum, spinal cord, and nerves.

*Inflammatory Softening of the Mucous Membranes.* Softening of the mucous membranes is a frequent occurrence, but more so in some than in others, and most of all in that of the digestive canal. Dr. Carswell justly observes that the alteration has assumed a high degree of importance in the latter, from the zealous attempt of the *école physiologique* to discover, in changes of the intestinal mucous membrane, the cause of the greater number of diseases. Softening of this portion of the mucous tissue occurs as a consequence of inflammatory action, or of that of the gastric juice. Our author first describes the former mode of alteration.

Softening of the mucous membrane of the stomach and intestines presents, in regard to degree and extent, the same variety as in the brain.

" In the first stage, the mucous membrane, instead of possessing that degree of cohesion peculiar to it in different portions of the alimentary canal, and which permits of its being detached from the submucous tissue in pieces of considerable size, breaks when seized between the fingers or forceps; in the second stage, the edge of a scalpel, or even the finger passed lightly over its surface, converts it into a soft, somewhat opaque, creamy-looking pulp; and in the third stage it is so completely softened and detached, that a gentle stream of water poured on it from the height of a few inches removes it entirely from the surface of the submucous tissue. The removal of portions of the mucous membrane of greater or less extent is effected in a similar manner during life, by the contents of the stomach and intestines in their passage through these organs. It is important to observe that in all these stages the softened mucous membrane is more or less

*opaque*; it does not present the *transparency* which accompanies softening from the chemical action of the gastric juice. In the former case, and in the last stage of softening, it resembles a mixture of flour and cold water or milk; in the latter, the same materials after having been submitted to the action of heat."

The extent of inflammatory softening of the mucous membrane is extremely variable. It has been described as involving the other tunics of the stomach and intestines, and producing perforation of these organs. But Dr. Carswell believes that this is always the consequence of the chemical action of the gastric juice.

The greater frequency of inflammatory softening in some portions than in others of the digestive mucous membrane is a circumstance deserving of attention, as it enables us to distinguish this alteration from that effected by the agency of the gastric juice. True inflammatory softening is *not* more frequent in the stomach than in the intestines. In the former, it occurs most frequently in the body and pyloric portion, whilst that which takes place at the fundus is most commonly effected by the gastric juice. The termination of the ileum, and the depending and fixed portions of the colon, as the cæcum, the right and left hypochondriac regions, and the sigmoid flexures of this gut, are the most frequent seats of inflammatory softening in the intestines.

" Lastly, inflammatory softening is more frequent in the follicular than in the villous structure of the mucous membrane. It is from this latter circumstance in particular that the *form* of inflammatory softening derives any diagnostic value. When the softening affects the glandulæ agminatæ and solitariæ, it presents the general form of these glands, viz. large oval, or small circular patches, and these are forms of softening which are always to be regarded as the result of a pathological cause, particularly if the surrounding mucous membrane does not present the same change, or any of the other physical characters of inflammation."

The principal varieties of colour of the softened mucous membrane are the red and pale. In the red softening, the tint may be confined to the softened part of the membrane, or it may extend to the other parts at the same time; or the latter may be red and the former pale. The redness may also be very slight or very considerable, and presents no peculiarity of form. In the *pale* softening, the mucous membrane presents a pale greyish, or yellowish grey tint, its natural colour being little altered; or it may be paler than natural, when it generally presents a milky aspect, owing to its being reduced to a thin layer, through which the colour of the submucous tissue is partially seen.

Inflammatory softening of the other mucous membranes does not differ in kind from that of the intestinal canal, and does not, therefore, need particular description. It is seldom considerable, except in the mucous membranes of the uterus, larynx, and bronchi, and is least of all in that of the urinary organs.

*Inflammatory Softening of the Cellular Tissue.* This is, in several respects a very important pathological lesion, the cellular tissue being by far the most frequent seat of softening; in consequence of which, inflamed parenchymatous organs, in general, are easily lacerated and broken down. This is well exemplified by the state of the lung in pneumonia.

Muscular tissue is also easily torn, or separated into shreds, in consequence of the softening of its interstitial cellular structure. This is often observed in the muscles of voluntary motion, and sometimes in the heart. The easy separation of the serous and mucous membranes is always the consequence of inflammatory softening of their connecting cellular tissue, and the degree of facility with which their separation is effected, affords a ready means of estimating the degree and extent of the inflammation to which this tissue had been subjected.

### Softening from Obliteration of Arteries,

This occurs only in the brain, and at an advanced period of life. Rostan described this as a disease sui generis, and compared it to gangrena senilis. The opinion has been strongly controverted, and it is singular that, out of the great number of cases of softening of the brain which M. Rostan relates, he has not given one in which ossification and obliteration of the arteries of the organ, are mentioned as having been observed at the autopsy.

The most essential of the physical characters of this lesion is the state of obliteration of the arteries. That obliteration may depend on various conditions of the vessel. Dr. Carswell conceives that the softening is the consequence of the cessation of nutrition, resulting from the impediment to the circulation.

The obliterated arteries may occupy the softened cerebral substance, and may be seen or felt ramifying through it. It may be so limited as to be overlooked. In the case of obliteration of minute arteries, or of a single small arterial trunk, the softening is generally confined to a space not exceeding an inch or two inches in superficial extent; but if several large contiguous branches be obliterated, the extent of the softening is considerably increased, or occupies two or more distinct portions of the brain ; and if the obliteration has taken place in the carotid or one of its principal divisions within the cranium, the greater part or the whole of a hemisphere may be softened.

Like inflammatory softening, this species is not confined to any particular portion of the brain, though it is more frequent in the brown than in the white substance, or in those parts most abundantly supplied with bloodvessels. Dr. C. has never observed this softening in the spinal cord. The degree of the alteration, as well as the colour, are often very similar to those observed in inflammatory softening. But redness is seldom considerable, and vascularity and effusion of blood are generally wanting, on account of the impervious state of the arteries. When effusion takes place, it is probably in consequence of rupture of the obliterated vessels, or of some of the smaller ones having remained pervious, and yielded to the increased momentum of the blood.

### Softening from the Chemical Action of the Gastric Juice.

Omitting Dr. Carswell's brief historical notice of this lesion, we are under the necessity of closely confining ourselves to his description. He observes that a number of experiments which he has made on healthy animals shew, that softening, erosion, and perforation of the digestive organs are produced, after death, in them, as in the human subject, by the dissolvent property of the gastric juice. The essential property of that juice, according to Dr. Carswell, is acidity; and, without that property, there is no dissolution of the stomach, intestines, nor other organs, after death.

"In every case of this lesion which I have observed, this property of the gastric juice was present, and was readily detected by its sour smell on laying open the stomach, or by means of litmus paper. That this, and no other, is the chemical cause of dissolution, is put beyond all doubt by *neutralizing* the gastric juice in the stomach of a healthy animal, killed during the act of digestion; for when this is done, *dissolution does not take place.* This, therefore, being the cause of the lesions I am about to describe, I shall use, in preference to the term gastric juice, that of gastric *acid.*"

Dr. Carswell proceeds to the enumeration and description of the physical characters of softening, &c. from the action of the "gastric acid" after death.

With respect to the situation of the lesion, it may be stated that it always occurs, or is greatest, at the most depending portion of the stomach. Under ordinary circumstances, this portion is the fundus, but accidental or other alterations in the neighbouring parts, or in the posture of the body, may modify it, and render other parts of the organ dependent.

"The degree of this kind of softening varies from a slight diminution of consistence of the coats of the stomach, to their conversion into a gelatinous pulp. In the first stage the mucous membrane (which is that which is first dissolved), presents a slight diminution of consistence, and has acquired a certain degree of transparency. When seized, it breaks immediately, or is crushed between the fingers into a soft pulp. In the second stage this membrane is seen lying like a quantity of albumen covering the submucous coat, and can be wiped off or removed by a bit of cloth or a stream of water. In the last stage the mucous membrane has entirely disappeared to a certain extent, thus leaving its submucous coat denuded, which is recognized by its grey silvery aspect. The same degrees of softening are observed in the other tunics of the stomach."

The extent of the alteration varies, being in one case confined to a small portion of the fundus, and, in another, occupying nearly the whole of the organ. It may be limited to the mucous membrane, or extend to all the tunics, when, perforation of the stomach occurring, the gastric acid escapes, acts upon the neighbouring organs—may perforate the diaphragm, and affect the contents of the chest, or pass into the œsophagus and dissolve its coats.

The form of this species of softening presents some important varieties.

"If the softening be confined to the mucous membrane of the fundus, the form which it assumes is that of small or large patches. These are generally irregular, their borders being formed by the mucous membrane, and the bottoms of each by the submucous coat; their edges, besides being irregular, are thin, soft, and somewhat transparent. If the softening has extended to the other coats of the stomach, the edges of these are beveled outwards, present a fringed appearance, or terminate in thin irregular prolongations, which, when water is poured upon them, are seen to float like shreds of transparent coagulable lymph. Such are the forms of softening of the mucous membrane, so long as this membrane is smooth or stretched out by the contents of the stomach. But when this membrane is thrown into folds, or forms plicæ, the softening occurs no longer in patches, but presents those remarkable appearances described by M. Louis, as indicating the existence of pathological alterations. The forms of the softening in this case are those of stripes and bands of various dimensions occupying the situation of the plicæ. Wherever these stripes and bands exist, we find that the mucous membrane has been completely dissolved and the submucous coat laid bare. They have thus a blueish or silvery grey aspect, while the mucous membrane which they enclose may be of its natural colour, red, brown, or yellow, and appears in isolated patches of various forms and extent. It was the isolated and defined character of this form of softening which made it be considered as indisputably of a pathological nature."

Dr. C. makes a few further not uninteresting remarks on the colour of this lesion, and the circumstances that prove its chemical origin. We regret that we have only room to recommend the Fasciculus to our readers.

